# Multicentric plasmacytic Castleman disease and IgG4-related disease manifested as cutaneous plasmacytosis: a report of two cases^☆^

**DOI:** 10.1016/j.abd.2026.501388

**Published:** 2026-06-16

**Authors:** Yao Yao, FengJie Liu

**Affiliations:** Department of Dermatology, Sun Yat-sen Memorial Hospital, Sun Yat-sen University, Guangzhou, China

Dear Editor,

Cutaneous plasmacytosis is a rare reactive lymphoproliferative disorder characterized by polyclonal plasma cell infiltration of the skin, often accompanied by systemic involvement, so that it is generally referred to as Cutaneous/Systemic Plasmacytosis (C/SP).^1^ It is most common among people aged 30–50 in East Asia, mainly Japanese, with a higher prevalence in men than in women.^1^ Its clinical manifestations include multiple reddish-brown plaques, and the axilla appears to be a typical and characteristic site of involvement, which may be a valuable clinical clue.^1^ Histopathology of skin lesions shows perivascular and periadnexal plasma cell infiltrates in the dermis.^2^ Extracutaneous involvement may occur most commonly in the superficial lymph nodes in about 58% of patients, and sometimes, the infiltrating cells form structures resembling the follicles of the lymph node.^2^ It shares overlapping clinical and histopathological features with Multicentric Plasmacytic Castleman Disease (MPCD) and IgG4-Related Disease (IgG4-RD), which both can cause plasma cell infiltration in the skin and systemic involvement, complicating differential diagnosis and therapeutic decision-making. Herein, we report two cases of East Asian males presenting with clinical manifestations consistent with cutaneous plasmacytosis, while the alternative diagnoses of MPCD and IgG4-RD were considered, respectively.

The first case is a 45-year-old man who presented with a 3-year history of progressive reddish-brown papules and nodules on the face, trunk, and axillae, forming infiltrative plaques along the dorsal midline (Fig. 1). Laboratory studies revealed polyclonal hypergammaglobulinemia (IgG 62.7 g/L [normal < 16]) and elevated inflammatory markers (IL-6, IL-2R, TNF-α). Ultrasound and Computed Tomography (CT) revealed enlarged lymph nodes in superficial and deep areas, including the cervical region, the porta hepatis, and the porta pulmonis. Skin biopsy showed dense perivascular and periadnexal plasma cell infiltration (Fig. 2) without light chain restriction or significant IgG4+ cells. The cervical lymph node biopsy revealed follicular hyperplasia with interfollicular sheets of CD138+ plasma cells (Fig. 3), and HHV-8 DNA testing in peripheral blood was negative, consistent with idiopathic Multicentric Plasma Cell Castleman Disease-Not Otherwise Specified (iMPCD-NOS). The patient received prednisone and siltuximab, a monoclonal antibody targeting IL-6, followed by a thalidomide-cyclophosphamide-prednisone regimen. The patient achieved partial remission six months after commencing siltuximab treatment, with rash and lymphadenopathy markedly subsiding and laboratory parameters improving.

**Fig. 1:**
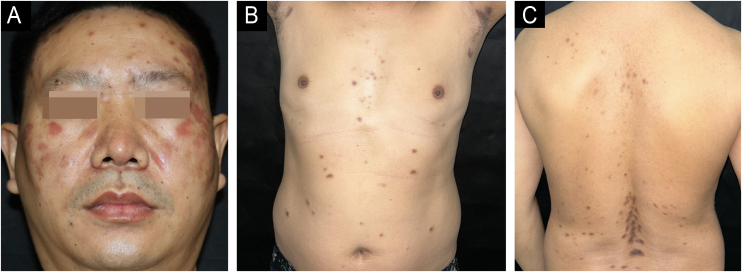
The skin lesions of Case 1 manifest as multiple dark red papules, nodules, and plaques on the face (A) with clear borders and mild infiltration, scattered brownish round patches on the trunk, and characteristic dorsal dark brown patches mainly on the axillae (B) and along the midspinal line (C).

**Fig. 2:**
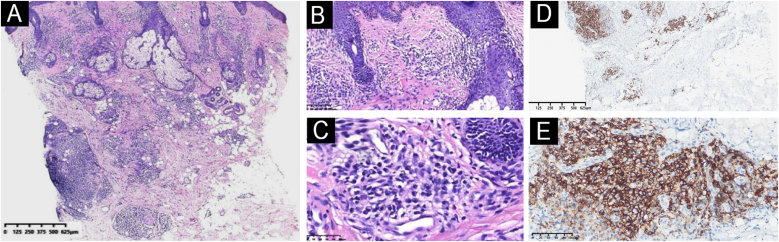
(A‒B) Skin biopsy shows intensive plasma cell infiltration in the whole layers of the dermis (A, Hematoxylin & eosin, ×40) and around vessels, appendages, and collagens (B, Hematoxylin & eosin, ×100). (C) Extensive perivascular plasma cell infiltration (Hematoxylin & eosin, ×400). (D‒E) Skin-infiltrating plasma cells show positive CD138 immunohistochemical staining (D ×40; E ×200).

**Fig. 3:**
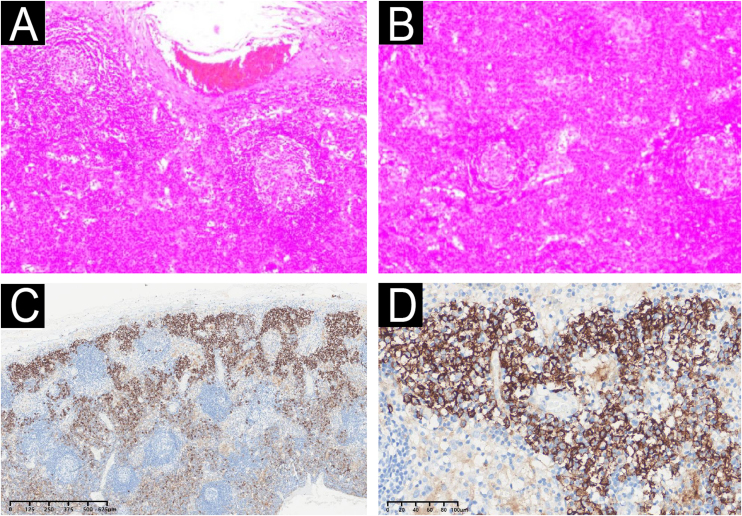
(A‒B) Histopathology of lymph nodes shows proliferation of lymphoid tissue, partial atrophy of lymphoid follicles, and considerable morphologically mature plasma cell infiltration between the follicles (Hematoxylin & eosin, ×100). (C‒D) CD138 immunohistochemical staining shows dense infiltration of CD138-positive plasma cells within lymphoid follicles (C ×40; D ×200).

The second patient, a 47-year-old man, presented with a 5-year history of infiltrative, dark-red papules and nodules localized to the left trunk (Fig. 4). Histopathological examination of an axillary lesion demonstrated dense dermal lymphoplasmacytic infiltrates surrounding vessels, adnexa, and nerves, composed of CD20 + B cells, CD79a + and CD138+ plasma cells, and scattered eosinophils (Fig. 5). The patient's serum IgG4 level was nearly 2-times the upper limit of normal. The lesion immunohistochemistry revealed that IgG4+ plasma cells constituted over 40% of IgG + plasma cells, with more than 10 IgG4+ plasma cells per high magnification field of view (Fig. 6). Ultrasound of the superficial lymph nodes revealed cervical lymphadenopathy. These clinicopathological manifestations are consistent with cutaneous plasmacytosis, but the high proportion of IgG4+ plasma cells in the skin lesions and elevated serum IgG4 level prompted us to consider the possibility of skin involvement of IgG4-RD. The patient declined cervical lymph nodes biopsy and treatment, and remained stable during 12-months of follow-up.

**Fig. 4:**
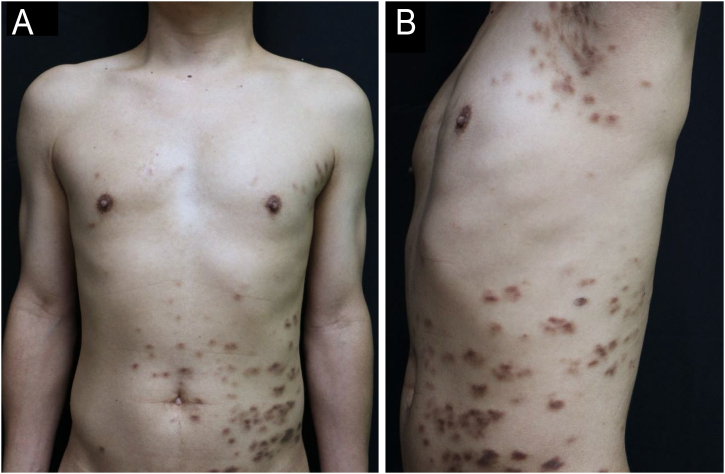
Multiple dark brown patches, papules, and nodules in the lumbar region and axillae (A‒B).

**Fig. 5:**
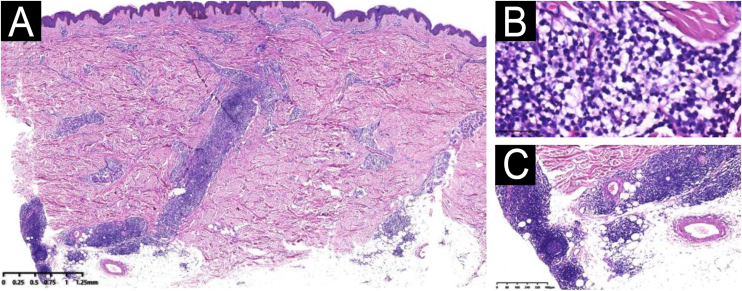
(A‒C) Histopathology shows dense infiltration of lymphocytes, plasma cells, and histiocytes in the dermal vasculature and the periphery of the small nerves and appendages (A, Hematoxylin & eosin, ×40; B, Hematoxylin & eosin, ×400; C, Hematoxylin & eosin, ×100).

**Fig. 6:**
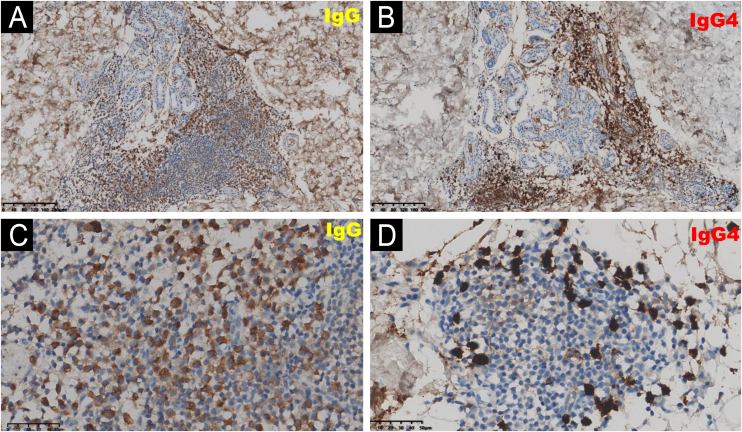
Skin lesion immunohistochemistry of case 2 showed IgG(+) and IgG4(+) plasma cell infiltration: (A, C) IgG + plasma cells (immunohistochemical staining for IgG, A ×100, C ×400). (B, D) IgG4+ plasma cells (immunohistochemical staining for IgG4, B ×100, D ×400).

C/SP and MPCD often share similar clinical manifestations, laboratory tests, and pathological changes. It has been suggested that C/SP is a variant of MPCD, or cutaneous MPCD,^3^ further complicating the relationship between them. However, several key differences may distinguish these two diseases. Firstly, regarding molecular mechanisms, high serum levels of IL-6 are frequently found in patients with MPCD and have been detected in some C/SP cases.^3^ IL-6 has a potentially similar and essential role in their pathogenesis, as it can induce the final differentiation of activated B-cells into immunoglobulin-producing cells.^3^ While the causes of elevated IL-6 in these two diseases differ. It was believed that the elevated IL-6 levels in MPCD were induced by HHV-8 infection, as the HHV-8 genome encodes a human IL-6 homolog,^4^ and almost half of HIV-negative MPCD cases were associated with HHV-8.^3^ In HHV-8-negative MPCD (iMPCD) patients, endogenous inflammation, paraneoplastic factors, or non-HHV-8 viruses (such as EBV, HBV, and HHV-6) may activate the NF-κB/JAK-STAT pathways, driving macrophages, stromal cells, and follicular dendritic cells to secrete IL-6, which establishes a cytokine storm and positive feedback loop for immune cell activation.^5^ The mechanism of elevated IL-6 in C/SP remains unclear. It has been reported that a point mutation in the proto-oncogene *c-kit* leads to constitutive activation of KIT, a tyrosine kinase, and that mutations in upstream signaling molecules may affect IL-6 activity in C/SP.^2^ Histopathologically, C/SP shows marked proliferation of mature polyclonal plasma cells in the cortical and medullary regions of the lymph nodes.^6^ Meanwhile, the enlarged lymph nodes of MPCD patients are usually small and distinguished by the presence of sheets of plasma cells in the interfollicular zone and hyperplastic germinal centres.^6^ Accordingly, the final diagnosis of MPCD rather than C/SP for the first case is mainly based on the lymph node pathological manifestations. From the perspective of disease progression, most patients with C/SP have chronic and benign courses, as MPCD exhibits a more aggressive and progressive development, and has a poorer prognosis, with 5-year survival rates ranging from only 55% to 77% reported in the literature.^7^ In conclusion, C/SP and MPCD are not essentially the same entity, and we should identify and definitively diagnose these two diseases early to apply different therapeutic strategies and assess their prognosis.

It has been reported that cutaneous plasmacytosis shares clinicopathologic overlaps with IgG4-RD^8^ and may represent a subtype of primary IgG4-related skin disease.^9^ Skin manifestations are rare in IgG4-RD, but skin biopsies are easier to perform and minimally invasive compared to those in IgG4-RD with other visceral organ involvement.^10^ A recent review suggested that skin lesions can be initial manifestations of systemic IgG4-RD.^10^ Early recognition of IgG4-RD is essential because delayed diagnosis and treatment may lead to irreversible organ fibrosis and damage. Collectively, these observations underscore the essential role of cutaneous manifestations in the early detection and definitive diagnosis of IgG4-RD, and long-term follow-up is warranted to monitor for the potential development of visceral and glandular involvement.

Although being considered as distinct disease entities, we should also recognize that partial spectral associations exist between C/SP, MPCD, and IgG4-RD. Some case reports indicate transitional states of them, such as elevated IgG4+ plasma cell proportions in C/SP patients or IgG4-RD pathological features in MPCD patients.^11^ Their core linkage lies in persistent immune dysregulation characterized by abnormal B-cell activation. Nevertheless, it remains crucial to note that differences in their pathogenic factors (C/SP and MPCD primarily involve IL-6,^5^ while IgG4-RD is dominated by IL-4/IL-21),^10^ core pathological features, treatment responses, and prognoses. In conclusion, C/SP, MPCD, and IgG4-RD may constitute a broad spectrum of immune-mediated plasma cell proliferative disorders that require multidimensional differential diagnosis.

Dermatologists should have a comprehensive understanding of cutaneous plasmacytosis and select diagnostic tests, such as light microscopy, immunohistochemistry, and serological testing, to determine whether it represents isolated C/SP or a cutaneous manifestation of another disease. Beyond MPCD and IgG4-RD, the clinical-pathological evaluation of C/SP must also consider the possibility of various tumors, infections, and connective tissue disorders to prevent misdiagnoses and treatment delays.

## Statement of ethics

Ethical approval is not required for this study in accordance with local or national guidelines. Written informed consent was obtained from the patient for publication of the details of their medical case and any accompanying images.

## Financial support

This work was supported by the Guangzhou Science and Technology Program Basic Research Program ‒ City School (Hospital) Jointly Funded Project ‒ Yat-Sen Youth Medical Talent Plan (Grant nº 2024A03J0917). The funders had no role in the design of the study, collection and analysis of data, drafting of the manuscript, or the decision to submit the manuscript for publication. The authors declare that they have no financial or non-financial conflicts of interest related to the funders or the content of this article.

## Authors' contributions

Liu Fengjie: Study conception and planning; manuscript critical review; intellectual participation in propaedeutic and/or therapeutic management of studied cases; effective participation in research orientation; critical literature review; approval of the final version of the manuscript.

Yao Yao: Data collection, analysis and interpretation; preparation and writing of the manuscript; manuscript critical review; critical literature review; intellectual participation in propaedeutic and/or therapeutic management of studied cases; effective participation in research orientation; approval of the final version of the manuscript.

## Research data availability

Does not apply.

## Conflicts ofinterest

None declared.
